# Supplementary material for: Causal Relationship between Aging and Anorexia Nervosa: A White-Matter-Microstructure-Mediated Mendelian Randomization Analysis
Source: Biomedicines. 2024 Aug 16;12(8):1874. doi: 10.3390/biomedicines12081874 (PMC11351342; doi:10.3390/biomedicines12081874)
Supplement: Supplementary file 1 [file biomedicines-12-01874-s001.zip › Supplementary Figure/Figure S7/Single SNP plot of MR analysis for ubm-b-1913 to AN.pdf]

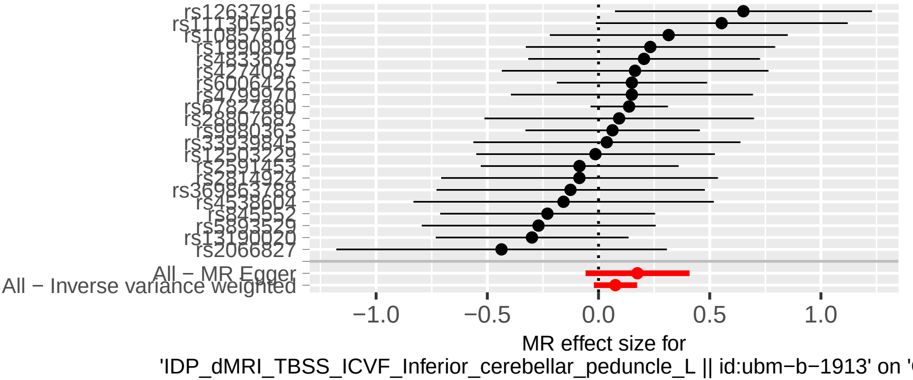
rs12637916
rs111305569
rs10857614
rs1990809
rs4833675
rs4274087
rs6006426
rs4799970
rs67827860
rs28807687
rs9980363
rs33939845
rs12503229
rs2591453
rs2814924
rs369863788
rs4538604
rs845552
rs5893529
rs13190020
rs2066827
All − MR Egger
All − Inverse variance weighted
−1.0
−0.5
0.0
0.5
1.0
MR effect size for
'IDP_dMRI_TBSS_ICVF_Inferior_cerebellar_peduncle_L || id:ubm−b−1913' on '
